# Modelling pacemaker oscillations in lymphatic muscle cells: lengthened action potentials by two distinct system effects

**DOI:** 10.1098/rsos.241714

**Published:** 2025-01-08

**Authors:** Edward J. Hancock, Charlie Macaskill, Scott D. Zawieja, Michael J. Davis, Christopher D. Bertram

**Affiliations:** ^1^ School of Mathematics & Statistics, University of Sydney, Sydney, New South Wales 2006, Australia; ^2^ Department of Medical Pharmacology & Physiology, University of Missouri, Columbia, MO 65212, USA

**Keywords:** calcium concentration oscillation, lymph transport, synchronized clocks, phase-plane analysis

## Abstract

Lymphatic system failures contribute to cardiovascular and various other diseases. A critical function of the lymphatic vascular system is the active pumping of fluid from the interstitium back into the blood circulation by periodic contractions of lymphatic muscle cells (LMCs) in the vessel walls. As in cardiac pacemaking, these periodic contractions can be interpreted as occurring due to linked pacemaker oscillations in the LMC membrane potential (M-clock) and calcium concentration (C-clock). We previously reported a minimal model of synchronized dual-clock-driven oscillations. While this qualitatively replicated the period of oscillations under different conditions, it did not replicate the action potential shape as it varied under those conditions, particularly as regards the extent or lack of a systolic plateau. Here, we modify the model to replicate the plateau behaviour. Using phase-plane analysis we show two qualitatively different dynamical mechanisms that could account for plateau formation, one largely M-clock-driven, the other largely C-clock-driven. The second case occurs with the introduction of a ryanodine receptor; in both cases, we find improved predictions for calcium levels. With enhanced fidelity to the experimental data, the improved model has the potential to help determine opportunities for pharmacological treatment of lymphatic system pumping defects.

## 1. Introduction

The lymphatic system consists of a network of vessels present in almost every organ of the body, plus the spleen, thymus and tonsils; bone marrow is also accounted a part of the system [1]. The vessels form a route for the return of excess interstitial fluid from all parts of the body to the subclavian veins, passing via lymph nodes. Node passage allows rapidly responding immunosurveillance of the body’s tissues for foreign matter, including bacteria and viruses. While immune cells at lymph nodes can identify and suppress tumour cells, notoriously the system’s defences can be overwhelmed or subverted, such that the lymphatic vascular system is a principal pathway for metastasis, as well as being implicated in numerous neurodegenerative and neuroinflammatory diseases [2].

Lymph is propelled along lymphatic vessels by one of two means; both rely on the subdivision of all the larger, collecting vessels into short segments by one-way valves. Extrinsic pumping takes place when collecting lymphatics are momentarily passively squeezed between surrounding tissues as a result of skeletal muscle use or other actions such as lung ventilation, blood vessel pulsation and gut peristalsis. Intrinsic pumping involves the periodic activation of muscle in the wall of collecting lymphatics to cause vessel constriction; the ejection fraction of a collector segment between two valves (termed a lymphangion [3]) can reach 80% [4]. Lymphatic muscle cells (LMCs) house their own inbuilt pacemaker [5], unlike the otherwise rather similar contractile cells of the gut wall [6].

We recently [7] published a model of LMC pacemaker oscillations that emulated biological findings from these cells under three different conditions: (i) control, simulating how the healthy LMC pacemaker behaves, (ii) simulating pharmacological or genetic ablation of anoctamin1 (Ano1) channel function, and (iii) simulating similar knock-out (KO) of inositol 1,4,5-triphosphate receptor (IP_3_R) function. Ano1 channels in the LMC membrane allow the release from the LMC of chloride ions down their concentration gradient, with the time-varying extent of channel opening mediated also by the intra-LMC free calcium-ion concentration [8]. After their blockade, the frequency of LMC contractions decreases sharply, and the LMC action potential (AP) becomes characteristically taller and shorter [9]. An earlier version of our model [10] was able to emulate these changes.

**Figure 1.**
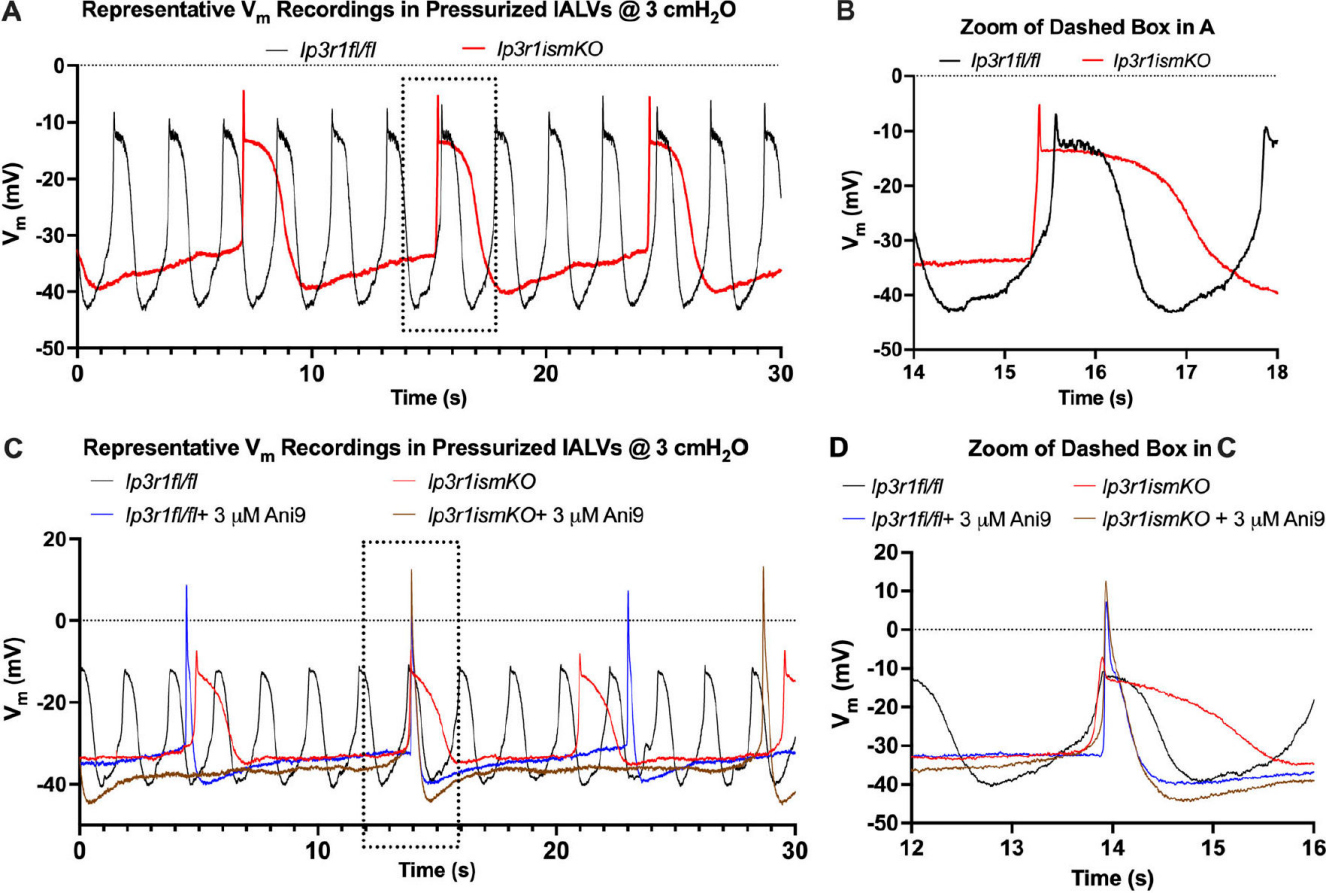
Representative recordings of membrane potential in murine inguinal-axillary LMCs, with the *ex vivo* vessel pressurized to 3 cmH_2_O. A and B compare control (black) and genetic IP_3_R-KO (red) conditions; C and D show different recordings of these same two conditions and also compare pharmacological Ano1-KO (blue) and combined Ano1-KO/IP_3_R-KO (brown) conditions. Figure compiled from figs 8A, 8B, 10A and 10B of Zawieja *et al*. [11], who retained copyright.

IP_3_ receptors in the membrane delimiting the LMC sarcoplasmic reticulum (SR) are the principal avenue for calcium-ion release from high concentration in the SR (also termed the store) to the cytoplasm [11]. This is a critical part of the lead-up to AP generation, because the [Ca^2+^]_c_ increase acts on the Ano1 channels, causing reduction in cytoplasmic [Cl^−^]. Both ionic changes cause the membrane potential to become less negative, thereby triggering the opening of voltage-gated L-type Ca^2+^ channels, allowing Ca^2+^ to flood into the cell from high concentration outside [12]. When IP_3_Rs are blocked, the frequency of APs decreases as much as during Ano1 blockade, but the remaining APs have a quite different shape from those seen under Ano1-KO, remaining of comparable height to those observed under control conditions but lasting longer, thanks to prolongation of the systolic plateau phase [11] (see figure 1).

**Figure 2.**
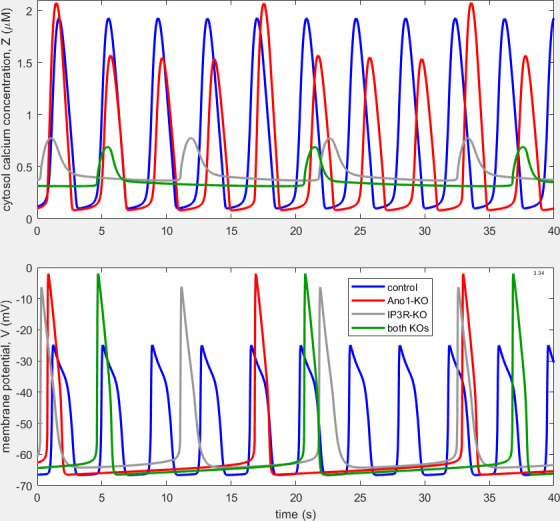
Waveforms of [Ca^2+^]_c_, $Z\left(t\right)$ (upper panel) and $V\left(t\right)$ (lower panel) under control (blue), Ano1-KO (red), IP_3_R-KO (grey) and combined-KO (green) conditions, from the model of Hancock *et al*. [7], long after start-up transients have decayed. See table 1 for parameter values. Apart from the combined-KO traces, these data were originally published in fig. 8 of Hancock *et al*. [7], who retained copyright.

We were able [7] to emulate the frequency reduction under IP_3_R-KO conditions by modifying both how the model specifies the characteristics of the IP_3_R and the model’s operating circumstances. The model follows established cardiac pacemaking practice (and terminology) in consisting of two linked ‘clocks’ [13]: one causing oscillation of the membrane potential (M-clock) and the other causing oscillation of calcium-ion concentration (C-clock), each in principle capable of oscillating independently. Formerly, when the model could emulate only control and Ano1-KO conditions [10], the parameter values chosen for the C-clock components were such that this clock could not spontaneously oscillate, instead merely responding in an oscillatory fashion to the periodic driving provided by the M-clock. We provided a conclusive analysis of this fact [7] and arranged our modified model such that both oscillators were positioned in their respective phase spaces at locations where they were unstable, i.e. each potentially oscillating independently until the two were entrained together by mutual coupling. Waveforms from that modified model are shown in figure 2.

However, the modified model still did not emulate satisfactorily all aspects of the observed IP_3_R-KO behaviour. The simulated IP_3_R-KO AP (figure 2) was tall and short, like that under Ano1-KO, and not even the control AP exhibited as much division into initial-spike and plateau phases as seen experimentally (figure 1). We also noted that the model and the experiments exhibited different patterns of cytosolic [Ca^2+^] oscillation: experimentally, Zawieja *et al*. [11] found that the waveform of [Ca^2+^]_c_ was at least as large in peak-to-trough excursion during IP_3_R-KO as under control conditions, whereas our model predicted much smaller waveform excursion during IP_3_R-KO. Furthermore, calibrated recordings of [Ca^2+^]_c_ waveforms in LMCs [16] show [Ca^2+^] oscillating between trough and peak limits of around 0.1 and 0.25−0.3 µM under control conditions,^1^ whereas the corresponding numbers for the model were 0.1 and 1.9 µM.

Addressing the issue of plateau length, we have now found that, with revised parameter values, the modified model is capable of allowing the AP in both control and IP_3_R-KO conditions to be well differentiated into initial-spike and plateau phases, with the IP_3_R-KO plateau emulating the experimental findings in being longer than the control one. The parameters modified are involved in Ca^2+^ fluxes, in the gating variable h for the L-type Ca^2+^ channels and in the Ca^2+^-induced Cl^−^ conductance (see details in table 1). However, these changes alone, while ameliorating, did not entirely remove the issue of relatively small [Ca^2+^]_c_ waveform excursion during IP_3_R-KO.

The need to maintain comparable [Ca^2+^]_c_ excursion under IP_3_R-KO was key to our discovery of a second route to a lengthened AP plateau. The relevant experiments had involved specifically knock-out of type 1 IP_3_ receptors, and it was determined that type 2 and type 3 IP_3_ receptors did not play a significant role in the LMC pacemaker. However, the process of investigating these possible parallel alternatives also led to consideration of the possible role of SR-membrane-located ryanodine receptors (RyRs). In cardiac myocytes, RyRs rather than IP_3_Rs are the principal avenue for Ca^2+^ release from the SR [18]; indeed, Ca^2+^ entry into the cell from outside via L-type channels is considered merely the trigger for the main biological mechanism of cytosolic [Ca^2+^] increase leading to contraction, via RyR calcium-induced calcium release (CICR). Arterial smooth muscle utilizes both mechanisms; smooth muscle excitability can be differentially regulated by either RyR or IP_3_R SR calcium release channels. But in LMCs, pacemaking is primarily driven by IP_3_R-mediated Ca^2+^ release [19]. Nevertheless, when the normally dominant IP_3_R1 Ca^2+^ flux was absent, it was clearly biologically possible that dormant RyRs might be taking over this job in this situation and thereby be responsible for maintaining [Ca^2+^]_c_ waveform excursion. In contrast, in the model, where the post-IP_3_R-KO [Ca^2+^]_c_ waveform was small, there was no representation of any alternative path for store Ca^2+^ release beyond passive leakage.

Accordingly we explored, and here describe, a further model variant in which RyRs are included. We find that the total excursion of the [Ca^2+^]_c_ waveform is much better maintained during IP_3_R-KO in this model. We also find that the changed [Ca^2+^]_c_ waveform, and in particular the increased peak height, brings with it a lengthening of the systolic plateau of the AP, and conversion of the AP to an overall shape and height that better matches what happens experimentally under IP_3_R-KO. By means of phase-plane analysis similar to that introduced in our previous papers [7,10], we show herein how these apparently distinct matters are inextricably linked, such that maximum [Ca^2+^]_c_ is a determinant of one dynamical mechanism of plateau formation and maintenance. We also provide a detailed analysis of the alternative dynamical mechanism of plateau formation which was identified in the model without RyRs.

## 2. Methods

Models of the biophysics of excitable muscle cells, if attempting to describe all ion species in play, can be very complicated: the model of the pacemaking interstitial cells of Cajal in the gut wall by Youm *et al*. [20] involves 23 differential equations, while the biophysical model of stretch responses in arterial smooth muscle cells by Karlin [21] has 63. In our models of the lymphatic pacemaker, we have espoused the alternative philosophy of keeping the model maximally simple, at the expense of detail, believing that a simple model can lead to greater insight than a more detailed one. In so doing, we can also take advantage of methods that are impractical for higher-dimensioned models, in particular phase-plane analysis.

Strictly, phase-plane analysis is possible only for models that have only two dimensions, i.e. are fully described by two ordinary differential equations. However, the identification of M- and C-clocks in the lymphatic pacemaker allowed a four-dimensional model [10], derived from that of Imtiaz *et al*. [12], that could be analysed on two phase planes, one each for the dependent variables of each clock, with the output of each clock influencing the other, when the coupling was not turned off for investigative purposes. That model was subsequently expanded [7] to five dimensions in order to explain IP_3_R-KO, with the C-clock becoming three-dimensional. However, to an adequate approximation, it remained possible to analyse the C-clock on a phase plane, since the time variation of one of its dependent variables was relatively small and almost linearly related to one of the other two.

Still being maximally parsimonious, we herein avoid expanding the dimensionality of the model further, by including a purely algebraic description of the action of RyRs taken from Keener & Sneyd [15]. The differential equations of the model are thus unchanged, as follows:


(2.1) $$\frac{dZ}{dt}=\frac{b_{\text{c}}}{v_{c}}\left(J_{se}-J_{si}-J_{ce}+J_{ci}-c\alpha I_{\text{L}}\right),$$

(2.2) $$\frac{dY}{dt}=\frac{b_{\text{c}}}{v_{\text{s}}}\left(-J_{se}+J_{si}\right),$$

(2.3) $$\frac{dn}{dt}=F_{1}Z\left(1-n\right)-F_{2}n,$$

(2.4) $$C_{\text{m}}\frac{dV}{dt}=\frac{1}{\alpha }J_{ci}-G_{\text{i}}\left(V-E_{\text{i}}\right)-G_{\text{C}}\left(V-E_{\text{C}}\right)-I_{\text{L}},$$

(2.5) $$\tau_{h}\frac{dh}{dt}=h_{\infty }-h.$$

The time-variables Z and Y are [Ca^2+^] in the cytosol and the store, respectively; n is an IP_3_R gating variable; V is the membrane potential; h is a gating variable for L-type Ca^2+^ channels; t is time. Functions on the right-hand side of these equations are (mostly nonlinear) algebraic constraints as follows:


(2.6) $$J_{\text{ce}}\left(Z\right)=\frac{J_{\text{xce}}Z^{2}}{K_{\text{ce}}^{2}+Z^{2}},$$

(2.7) $$J_{\text{ci}}\left(P\right)=A_{1}+A_{2}v_{\text{c}}P,$$

(2.8) $$p_{\text{ip}}\left(Z,n,P\right)={\left(\frac{PZ\left(1-n\right)}{\left(P+K_{1}\right)\left(Z+K_{5}\right)}\right)}^{3},$$

(2.9) $$J_{\text{ip}}\left(Z,Y,n,P\right)=k_{\text{f}}p_{\text{ip}}\left(Y-Z\right),$$

(2.10) $$p_{\text{ry}}\left(Z\right)=\frac{Z^{\rho }}{K_{\text{r}}^{\rho }+Z^{\rho }},$$

(2.11) $$J_{\text{ry}}\left(Z,Y\right)=k_{\text{r}}p_{\text{ry}}\left(Y-Z\right),$$

(2.12) $$J_{\text{lk}}\left(Z,Y\right)=k_{\text{se}}\left(Y-Z\right),$$

(2.13) $$J_{\text{se}}\left(Z,Y,n,P\right)=J_{\text{ip}}+J_{\text{ry}}+J_{\text{lk}},$$

(2.14) $$J_{\text{si}}\left(Z\right)=\frac{J_{\text{xsi}}Z^{2}}{K_{s\text{i}}^{2}+Z^{2}},$$

(2.15) $$F_{1}\left(P\right)=\frac{k_{\text{m}4}K_{1}K_{2}+k_{\text{m}2}K_{4}P}{K_{4}K_{2}\left(P+K_{1}\right)},$$

(2.16) $$F_{2}\left(P\right)=\frac{k_{\text{m}2}P+k_{\text{m}4}K_{3}}{P+K_{3}},$$

(2.17) $$K_{i}=\frac{k_{\text{m}i}}{k_{\text{p}i}}\;for\;i=1,\ldots ,5,$$

(2.18) $$\alpha =\frac{1}{zF},$$

(2.19) $$G_{\text{C}}\left(Z\right)=G_{\text{xc}}\frac{Z^{q}}{K_{\text{C}}^{q}+Z^{q}},$$

(2.20) $$I_{\text{L}}\left(V,h\right)=G_{\text{L}}mh\left(V-E_{\text{L}}\right),$$

(2.21) $$m\left(V\right)=\frac{1}{1+\exp\left(\frac{V-V_{\text{hm}}}{V_{\text{km}}}\right)},$$

(2.22) $$h_{\infty }\left(Z,V\right)=\frac{K_{\text{h}}^{\psi }}{K_{\text{h}}^{\psi }+Z^{\psi }}\frac{1}{1+\exp\left(\frac{V-V_{\text{hh}}}{V_{\text{kh}}}\right)},$$

(2.23) $$\tau_{\text{h}}\left(V\right)=\tau_{\text{hn}}+\left(\tau_{\text{hx}}-\tau_{\text{hn}}\right)\frac{1}{1+\exp\left(\frac{V-V_{\text{h}\text{t}}}{V_{\text{k}\text{t}}}\right)}.$$

Most of the equations (2.6)−(2.23) are also unchanged from [7]; the addition of RyRs is represented in equations (2.10) and (2.11), where equation (2.10), adapted from [15], is a simple model of Ca^2+^-induced Ca^2+^ release (CICR). Also, the constant $V_{\text{h}\text{t}}$ in equation (2.23) is now distinct from $V_{\text{h}\text{h}}$ in equation (2.22), where previously it was not. In equations (2.6)−(2.23), the left-hand-side quantities are defined as follows. $J_{\text{ci}}$ and $J_{\text{ce}}$ are the calcium-ion fluxes into and out of the cell^2^ (excluding influx via L-type channels); $J_{\text{si}}$ and $J_{\text{se}}$ are the equivalent fluxes into and out of the store. $J_{\text{se}}$ consists of the fluxes $J_{\text{ip}}$ via IP_3_Rs, $J_{\text{ry}}$ via RyRs and $J_{\text{lk}}$ by leakage. The probability of the IP_3_Rs and RyRs being open is given by $p_{\text{ip}}$ and $p_{\text{ry}}$, respectively. $F_{1}$ and $F_{2}$ are functions used in equation (2.3); their values (as with many of the fluxes) depend on P, the intracellular concentration of IP_3_, shown as a possible time-variable but actually held constant in what we report here.^3^ α, used in equations (2.1) and (2.4), is a conversion factor for the current created by a calcium-ion flux. $G_{\text{C}}$, used in equation (2.4), is the Z-dependent conductance of Ano1 Cl^−^ channels.

$I_{\text{L}}$ is the current via L-type Ca^2+^ channels, controlled by the gating variables h and m, with the latter assumed time-independent [12]; $h_{\infty }$ is the Z- and V-dependent value towards which h decays exponentially at a rate which is controlled by the V-dependent time constant $\tau_{\text{h}}$. The definitions and values of the constants that appear on the right-hand side of all the above equations are given in table 1.

As in our previous published paper [7], the theoretical complete absence of the target genes and their encoded proteins was modelled in the case of Ano1-KO [10] by setting $G_{\text{xC}}$ = 0, and in the case of IP_3_R-KO [7], by setting $k_{\text{f}}$ = 0; for combined Ano1-KO and IP_3_R-KO, both $G_{\text{xC}}$ and $k_{\text{f}}$ were set to zero. See Zawieja *et al*. [9,11] for details of the corresponding experimental procedures.

To obtain the results shown in the next section, finite-difference versions of the equations of the model were implemented in Matlab version R2019b (The Mathworks, Natick, MA, USA) and integrated over time using the Matlab variable-order solver ode15s for stiff differential/algebraic systems. Matlab code for the purpose is available via a public repository (see Data Accessibility), and phase-plane animations are available as electronic supplementary material.

## 3. Results

### 3.1. Model without RyRs

We herein present results that represent a qualitatively novel study of plateau behaviour. We first show results from a model having the equations used by Hancock *et al*. [7] but with parameter values chosen (after exhaustive searching) so as to maximize the duration of the AP plateau under IP_3_R-KO, without losing the existing data-emulating waveform characteristics under control and Ano1-KO conditions. The aim is thus to emulate the major qualitative ways in which the biological AP changes when IP_3_ receptors are blocked, namely, reduced frequency, lengthened systole and peak-to-trough excursion comparable in magnitude with corresponding control APs (figure 1). Explicitly, we have modified the values of parameters $J_{\text{xse}}$ and $J_{\text{xci}}$, which determine the Ca^2+^ fluxes given in equations (2.6) and (2.14), respectively, $k_{\text{se}}$ and $k_{\text{f}}$ in the fluxes given by equations (2.9) and (2.12), the three parameters on the right-hand side of equation (2.19) for the conductance $G_{\text{C}}$ of Ano1 Cl^−^ channels, and finally parameters in equations (2.22) and (2.23) for $h_{\infty }$ and $\tau_{\text{h}}$.

**Table 1.** Values for constants in the model. Sources (otherwise, estimated): 1 is Imtiaz *et al*. [12], although in many cases the values have been reinterpreted in terms of units, etc., 2 is Lees-Green *et al*. [14], 3 is Keener & Sneyd [15] and 4 is a universal physical constant. Estimated values were based on existing sources.

parameter	units	meaning	figure 2 value	figure 3 value	figure 4 value	source
$J_{\text{xce}}$	µmol s^−1^	max. Ca^2+^ efflux from cell via pmCa pump	0.28$v_{c}/b_{c}$	0.56$v_{c}/b_{c}$	0.28$v_{c}/b_{c}$	
$K_{\text{ce}}$	µM	half-saturation *Z*-value for pmCa pump	0.425	0.425	0.425	
$A_{1}$	µmol s^−1^	constant Ca^2+^ leak into cell	0.0015$v_{c}/b_{c}$	0.0015$v_{c}/b_{c}$	0.0015$v_{c}/b_{c}$	
$A_{2}$	s^−1^	Ca^2+^ flux into cell per unit of IP_3_	0.01$/b_{c}$	0.01$/b_{c}$	0.01$/b_{c}$	
P	µM	IP_3_ concentration in cytoplasm	0.7	0.7	0.7	
$J_{\text{xsi}}$	µmol s^−1^	maximum SERCA flux	9$v_{c}/b_{c}$	1.62$v_{c}/b_{c}$	1.62$v_{c}/b_{c}$	
$K_{\text{si}}$	µM	half-saturation *Z*-value for SERCA pump	0.1	0.1	0.1	
$k_{\text{se}}$	l s^−1^	constant Ca^2+^ leak from store	3.3$v_{c}/b_{c}$	0.04$v_{c}/b_{c}$	0.04$v_{c}/b_{c}$	
$k_{\text{f}}$	l s^−1^	scale for IP_3_-dependent store Ca^2+^ efflux	4.8$v_{c}/b_{c}$	0.96$v_{c}/b_{c}$	0.96$v_{c}/b_{c}$	
$k_{\text{p}1}$	/µmol s^−1^	IP_3_R binding constant	400	400	400	3
$k_{\text{p}2}$	/µmol s^−1^	IP_3_R binding constant	0.2	0.2	0.2	3
$k_{\text{p}3}$	/µmol s^−1^	IP_3_R binding constant	400	400	400	3
$k_{\text{p}4}$	/µmol s^−1^	IP_3_R binding constant	0.2	0.2	0.2	3
$k_{\text{p}5}$	/µmol s^−1^	IP_3_R binding constant	20	20	20	3
$k_{\text{m}1}$	s^−1^	IP_3_R binding constant	52	52	52	3
$k_{\text{m}2}$	s^−1^	IP_3_R binding constant	0.21	0.21	0.21	3
$k_{\text{m}3}$	s^−1^	IP_3_R binding constant	377.2	377.2	377.2	3
$k_{\text{m}4}$	s^−1^	IP_3_R binding constant	0.029	0.029	0.029	3
$k_{\text{m}5}$	s^−1^	IP_3_R binding constant	1.64	1.64	1.64	3
$K_{r}$	µM	half-saturation *Z*-value for RyRs	—	—	0.4	
$k_{\text{r}}$	l s^−1^	scale for store Ca^2+^ efflux via RyRs	—	—	0.0384$v_{c}/b_{c}$	
ρ	—	Hill-function exponent for $J_{\text{ry}}\left(Z,Y\right)$	—	—	2	
$b_{\text{c}}$	—	Ca^2+^ buffering constant	0.01	0.01	0.01	2
$v_{\text{c}}$	l	cytosol volume	10^−12^	10^−12^	10^−12^	2
$v_{\text{s}}$	l	store volume	$v_{c}$/5.5	$v_{c}$/5.4	$v_{c}$/5.5	3
$C_{\text{m}}$	pF	cell capacitance	25	25	25	2
$E_{\text{i}}$	mV	reversal potential, non-selective channels	−67.2	−67.2	−67.2	1
$E_{\text{C}}$	mV	reversal potential for Ano1 channels	−20	−20	−20	1
$E_{\text{L}}$	mV	reversal potential for L-type channels	+20	+20	+20	1
$G_{\text{i}}$	nS	conductance of passive channels	0.4	0.4	0.4	
$G_{\text{xC}}$	nS	max. Ca^2+^-induced Cl^−^ conductance	0.8	0.15	0.7	
$G_{\text{L}}$	nS	conductance of L-type Ca^2+^ channels	1.7	1.7	1.7	
$K_{\text{C}}$	µM	half-saturation *Z*-value for $G_{\text{C}}\left(Z\right)$	1.1	0.25	0.7	
q	—	Hill-function exponent for $G_{\text{C}}\left(Z\right)$	4	1	2	
$V_{\text{hm}}$	mV	half saturation for gate fn. $m\left(V\right)$	−46	−46	−46	
$V_{\text{km}}$	mV	inverse slope const., gate fn. $m\left(V\right)$	−4	−4	−4	
$K_{\text{h}}$	µM	half saturation for gate fn. $h_{\infty }\left(Z\right)$	4	0.25	0.4	
ψ	—	Hill-function exponent for $h_{\infty }\left(Z\right)$	20	20	4	
$V_{\text{hh}}$	mV	half saturation for gate fn. $h_{\infty }\left(V\right)$	−48	−40	−45	
$V_{\text{kh}}$	mV	inverse slope const., gate fn. $h_{\infty }\left(V\right)$	1	1	1	
$\tau_{\text{hn}}$	s	minimum of time constant $\tau_{\text{h}}\left(V\right)$	0.5	0.3	0.3	
$\tau_{\text{hx}}$	s	maximum of time constant $\tau_{\text{h}}\left(V\right)$	10	20	20	
$V_{\text{ht}}$	mV	half saturation for time const. $\tau_{\text{h}}\left(V\right)$	= $V_{\text{hh}}$	= $V_{\text{hh}}$	−40	
$V_{\text{kt}}$	mV	inverse slope const., time const. $\tau_{\text{h}}\left(V\right)$	= $V_{\text{kh}}$	= $V_{\text{kh}}$	= $V_{\text{kh}}$	
c	—	binary switch to block $Z\left(I_{\text{L}}\right)$	1	1	1	
α	(µmol s^−1^) pA^−1^	Ca^2+^ flux per unit current	5.18 ⨯ 10^−12^	5.18 ⨯ 10^−12^	5.18 ⨯ 10^−12^	4
z	—	valency of calcium ion	2	2	2	4
F	pC µmol^−1^	Faraday constant	9.6485 ⨯ 10^10^	9.6485 ⨯ 10^10^	9.6485 ⨯ 10^10^	4

**Figure 3.**
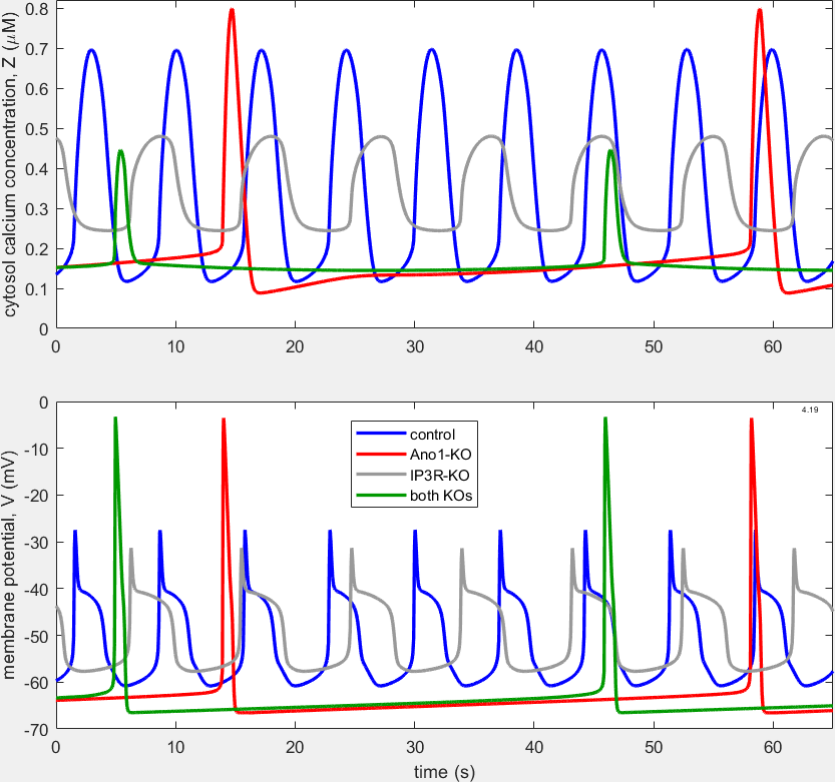
Waveforms of $Z\left(t\right)$ (upper panel) and $V\left(t\right)$ (lower panel) under control (blue), Ano1-KO (red), IP3R-KO (grey) and combined-KO (green) conditions for the model of Hancock *et al*. [7] after transient decay, but with parameters set up to maximize IP_3_R-KO plateau formation. The AP frequencies are 8.43, 1.36, 6.48 and 1.46 min^−1^ for control, Ano1-KO, IP_3_R-KO and combined-KO conditions, respectively.

Figure 3 shows the resulting waveforms of intracellular [Ca^2+^], $Z\left(t\right)$, and membrane potential, $V\left(t\right)$. As in figure 2, four different simulations are overlaid in each panel, representing control conditions (blue trace), Ano1 KO (red trace), IP_3_R KO (grey trace) and combined Ano1 and IP_3_R KO (green trace). Whilst in the waveforms shown in [7] the APs under IP_3_R-KO had the same morphology as those under Ano1-KO (figure 2), now the IP_3_R-KO APs have approximately the same shape as those under control conditions, i.e. a pronounced (even exaggerated^4^) initial spike and a subsequent plateau. Despite the greater duration of the control APs, relative to those in figure 2, the IP_3_R-KO APs are longer still, as observed biologically (figure 1). As required, the frequency of IP_3_R-KO APs is less than that of control APs, although, at 77% of the control frequency, not as much less as seen experimentally—some 38% of control (see fig. 8 of [10])—but control frequency itself varies widely between specimens at a given pressure (see fig. 3 of [11]). In comparison with the excursion magnitude of IP_3_R-KO APs obtained formerly (57.9 mV), which was almost as large (89.8%) as that of Ano1-KO APs (64.5 mV), and 38.9% larger than that of control APs (41.7 mV), now the IP_3_R-KO AP excursion magnitude (26.3 mV) is 21% smaller than the control AP excursion (33.3 mV). Furthermore, whilst previously the IP_3_R-KO excursion of $Z\left(t\right)$ was only 22.2% of control, now it has increased to 41% of control, and this improvement is achieved with the reduction of the control Z-excursion to 0.58 µM, much closer to that measured experimentally [16].

Panels C and D of figure 1 also provide data on the biological AP under simultaneous blockade of both Ano1 (pharmacologically) and IP_3_R (genetically). The AP is similar in shape to that under Ano1-KO, but the initial spike is taller and the subsequent hyper-polarization is greater, while the frequency is slightly further depressed (not evident in figure 1, but shown to be significant at the level *p* < 0.05 [11]). The model of Hancock *et al*. [7] in contrast predicted essentially the same values for the double-KO AP peak and trough as for Ano1-KO by itself, and, likewise, unchanged frequency. The model, as shown here in figure 3, again predicts essentially unchanged AP peak and trough values for double-KO versus Ano1-KO, but double-KO frequency is predicted to be 7.7% greater than Ano1-KO frequency.

### 3.2. Model with RyRs

**Figure 4.**
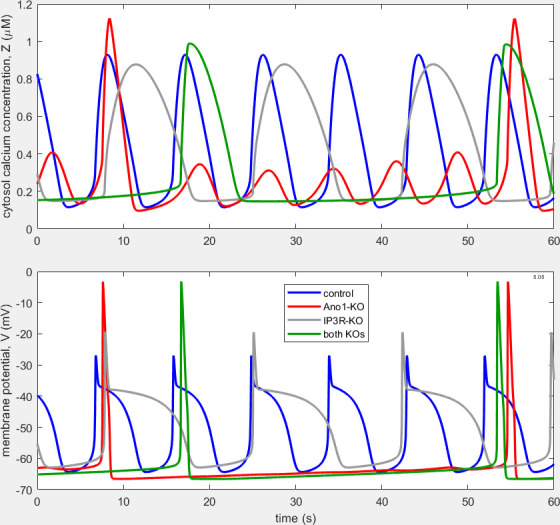
Waveforms as in figure 3 of $Z\left(t\right)$ and $V\left(t\right)$ under control, Ano1-KO, IP_3_R-KO and combined-KO conditions in the model with included RyR, after transient decay. The frequency of APs is 6.64, 1.27, 3.47 and 1.56 min^−1^, respectively. With these parameter values (table 1), the involvement of RyR is essential; if it is blocked, oscillation ceases under control and IP_3_R-KO conditions, and the loss of RyR cannot be compensated by artificially increasing store leakage.

With the twin modifications of the RyR and a separate value for $V_{\text{h}\text{t}}$, along with minor adjustments to some of the other parameter values that were previously adjusted to obtain figure 3 (see table 1), it proved possible to obtain further improvement in the degree to which the model waveforms emulate those observed by Zawieja *et al*. [11] (see figure 4). The AP under IP_3_R-KO is now approximately twice as long as the control AP. In addition, the duration of the diastolic slow depolarization phase is now greater than control; together, these changes mean that the frequency of APs under IP_3_R-KO is now only 52% of that under control conditions. Furthermore, the IP_3_R-KO Z-excursion (0.73 µM) is now 90% of the control Z-excursion (0.81 µM), although both are larger than what is observed physiologically. The IP_3_R-KO V-excursion (43.4 mV) is now 16% larger than control (37.4 mV), whereas in the example recordings shown in figure 1, in which the initial AP spike is relatively smaller, the IP_3_R-KO V-excursion is mostly slightly less than control. Combined Ano1-KO and IP_3_R-KO produce APs that are essentially identical to those under Ano1-KO alone, although they occur 22.7% more frequently. The similarity of APs under Ano1-KO and under combined-KO in the model matches the behaviour seen biologically in panel D of figure 1.

In all three models considered (figures 2−4), control APs are shifted negatively by some 15−20 mV relative to those seen in figure 1. Table 2 compares all three models with experimental data in terms of frequency, AP width and peak-to-trough size for V and Z.

**Table 2.** Comparison of experiments with model outputs, in terms of AP frequency, AP width and peak-to-trough size for V-waveforms and Z-waveforms. All numbers are percentages, showing the ratio of the relevant measurement under one of the three challenges (Ano1-KO, IP3R-KO, both KOs) to the corresponding measurement under control conditions. The experimental data are partly from Zawieja *et al*. [9,11], each measurement being the mean from several experiments. All Ano1-KO and both-KO experimental data involve the use of the pharmacological agent Ani9 [22], not genetic KO animals. Experimentally, AP width was measured as described by Zawieja *et al*. [23]; in the models, it was measured at a level one-quarter of the way from $V_{\text{min}}$ to $V_{\text{max}}$.

		experiments	figure 2	figure 3	figure 4
Ano1-KO/control	frequency	31	24	16	19
	$V_{\text{max}}-V_{\text{min}}$	146	155	189	169
	AP width	42	65	32	19
	$Z_{\text{max}}-Z_{\text{min}}$	223	109	123	126
IP3R-KO/control	frequency	33	36	77	52
	$V_{\text{max}}-V_{\text{min}}$	76	139	79	116
	AP width	179	77	144	206
	$Z_{\text{max}}-Z_{\text{min}}$	121	22	41	90
both-KO/control	frequency	27	24	17	24
	$V_{\text{max}}-V_{\text{min}}$	145	155	190	169
	AP width	50	65	33	19
	$Z_{\text{max}}-Z_{\text{min}}$	—	21	52	103

### 3.3. Plateau formation without RyR

**Figure 5.**
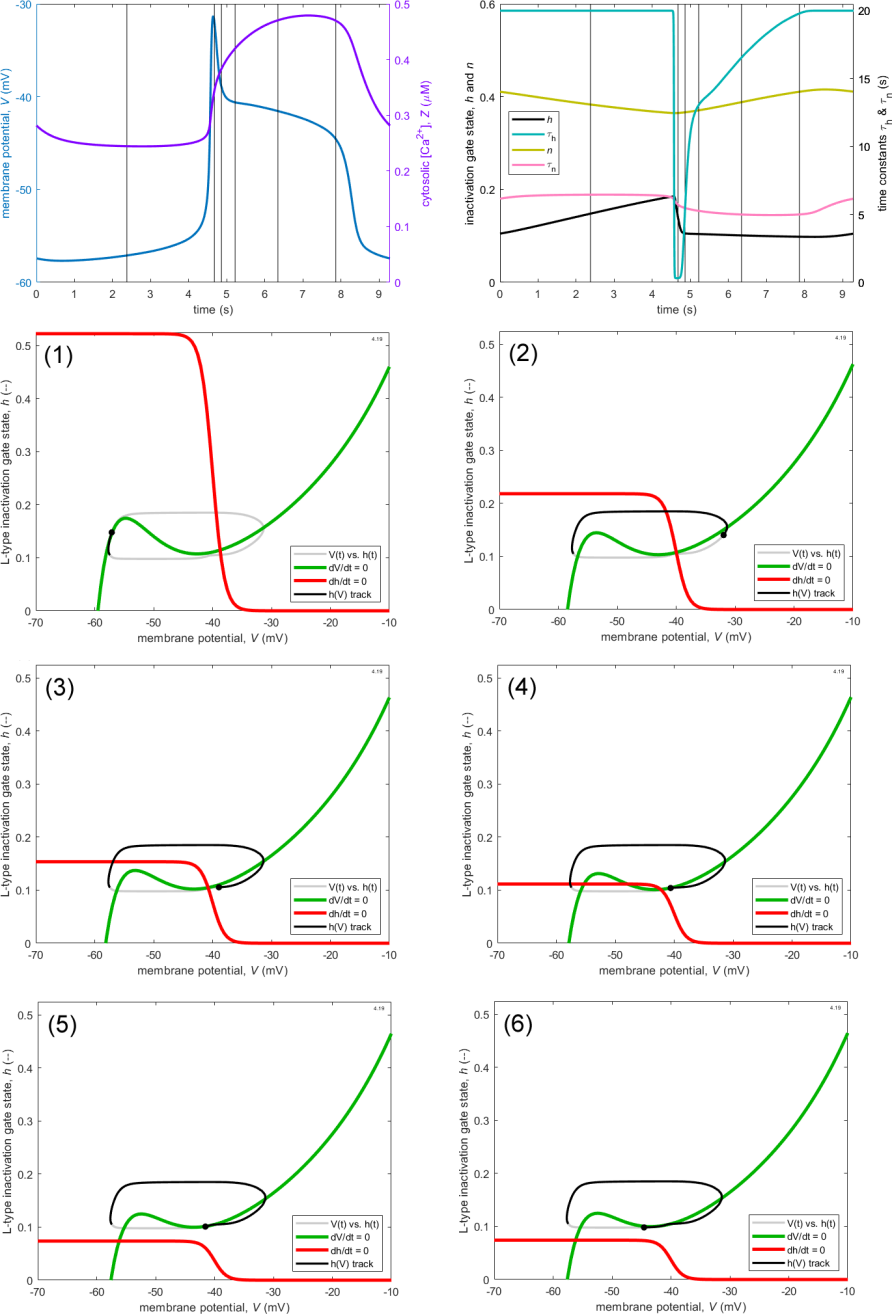
The M-clock (V, h)-phase plane for the pacemaker oscillation under IP_3_R-KO shown in figure 3, at the six instants shown by vertical lines in the top two panels, which show the time-course of $V\left(t\right)$ and $Z\left(t\right)$ (left panel) and the time-course of $h\left(t\right)$, $n\left(t\right)$, $\tau_{\text{h}}\left(t\right)$ and $\tau_{\text{n}}\left(t\right)$ (right panel). Besides the track of $h\left(V\right)$ (black curve and point on grey loop), the phase planes also show the dynamically varying position of the curves (nullclines), where dV/dt = 0 (green) and dh/dt = 0 (red).

We now turn to analyse the reasons for the production of the systolic plateau in the model, this being such a prominent feature of the experimentally observed IP_3_R-KO APs. We find that the dynamical mechanism of plateau production is qualitatively subtly different between the two models introduced here (figures 3 and 4). The analysis requires consideration of the M-clock phase plane, i.e. that determined principally by equations (2.4) and (2.5). Figure 5 shows the (V, h) phase space at six different salient instants during one complete cycle of the oscillation depicted in figure 3. The six times are shown by vertical lines in the top panels of figure 5. These panels show, on the left, the AP and the corresponding time variation of cytosol [Ca^2+^] and, on the right, the simultaneous variation of the gating variables h and n, and of their respective time-constants $\tau_{\text{h}}$ and $\tau_{\text{n}}$.^5^

The phase space is divided into two regions for each of the axis variables V and h, by a nullcline where the corresponding variable does not change with time. The nullcline is the zero contour of a map of the rate of change of the variable. On one side of the nullcline, the time-derivative of said variable is positive; on the other side, it is negative. For any useful phase space, the sides will be arranged such that the operating point moves towards the nullcline, and in this sense, each nullcline can be thought of as attracting,^6^ along a line parallel to its corresponding axis. In this respect, the nullclines can be said to organize the phase space. Note that the position of the nullclines itself varies over time.

Nullcline position is here controlled by the instantaneous value of Z; for the h-nullcline (red curve), which is simply $h_{\infty }$, the first term of the product on the right-hand side of equation (2.22) shows that as Z increases, the maximum of the h-nullcline gets smaller. The dependence of the V-nullcline on Z is less straightforward to see, but basically, as Z increases, the V-nullcline curve moves downwards and to the right, while changing shape. This motion was illustrated by Hancock *et al*. [10]—their fig. 4*c*). Nullcline attraction is parallel to the relevant axis (horizontal for V and vertical for h) and generally increases with distance (parallel to that same axis) from the nullcline.

In figure 5, the six instants to be discussed are identified in the phase planes as (1)−(6); in what follows, we will refer to times $t_{1}$ to $t_{6}$. In diastole, as the operating point climbs the left-hand limb of the M-clock orbital trajectory ($t_{1}$), the dynamics of h are relatively slow, because $\tau_{\text{h}}$ is large (20 s). This is despite the fact that at this time the left-hand part of the h-nullcline (red curve) is far above the operating point (black dot), and so $h_{\infty }-h$ (the vertical distance between the h-nullcline and the operating point) is quite large; recall that $dh/dt=\left(h_{\infty }-h\right)/\tau_{\text{h}}$. Conversely, once the operating point has passed the peak of the AP and started to descend the right-hand limb of the M-clock orbit ($t_{2}$), $\tau_{\text{h}}$ is at its minimum, and so the dynamics of h are fast: h (and V) reduce quickly. This effect outweighs the fact that $h_{\infty }-h$ is much less than at $t_{1}$.

However, as the operating point approaches the end of the early AP spike ($t_{3}$), the numerator and the denominator of the expression for dh/dt combine to slow things down. Both effects result from the operating point now being above the sloping portion of the h-nullcline, so that $h_{\infty }-h$ is small and reducing. Since the instantaneous membrane potential V also defines $\tau_{\text{h}}$, via a curve that is qualitatively identical to the h-nullcline, $\tau_{\text{h}}$ is now rapidly increasing.

As the operating point, having moved further to the left in its M-clock orbit, moves onto the start of the AP plateau ($t_{4}$), $\tau_{\text{h}}$ is now very substantially elevated (≈13 s) and $h_{\infty }-h$ is further reduced. At this time, h is close to (but not yet at) its minimum, and, importantly, the local minimum of the V-nullcline (green curve) is below the operating point. Thus the way is not yet open for the operating point to be attracted to the stable equilibrium offered by the left-hand limb of the V-nullcline. Nor is progress in that direction possible while the operating point is below the left-hand part of the h-nullcline.

As the operating point arrives at the mid-point of the AP plateau ($t_{5}$), the V-nullcline continues to block the route back to the resting potential, but the h-nullcline has withdrawn (because Z is approaching its peak value). However, with the operating point defining an ever-increasing value of $\tau_{\text{h}}$ (now up to ≈17 s), even the very small further h-descent that would suffice to clear the V-nullcline minimum and bring the AP to an end is going to have to take a relatively long time (almost 2 s more).

Finally, with h arriving at its minimum, the V-nullcline minimum is cleared ($t_{6}$), and the operating point accelerates towards the left-hand limb of the V-nullcline, i.e. the plateau phase merges into the rapid descent phase of the AP. No further h-descent is needed, so the continuing slow h-dynamics become irrelevant.

### 3.4. Plateau formation with RyR

**Figure 6.**
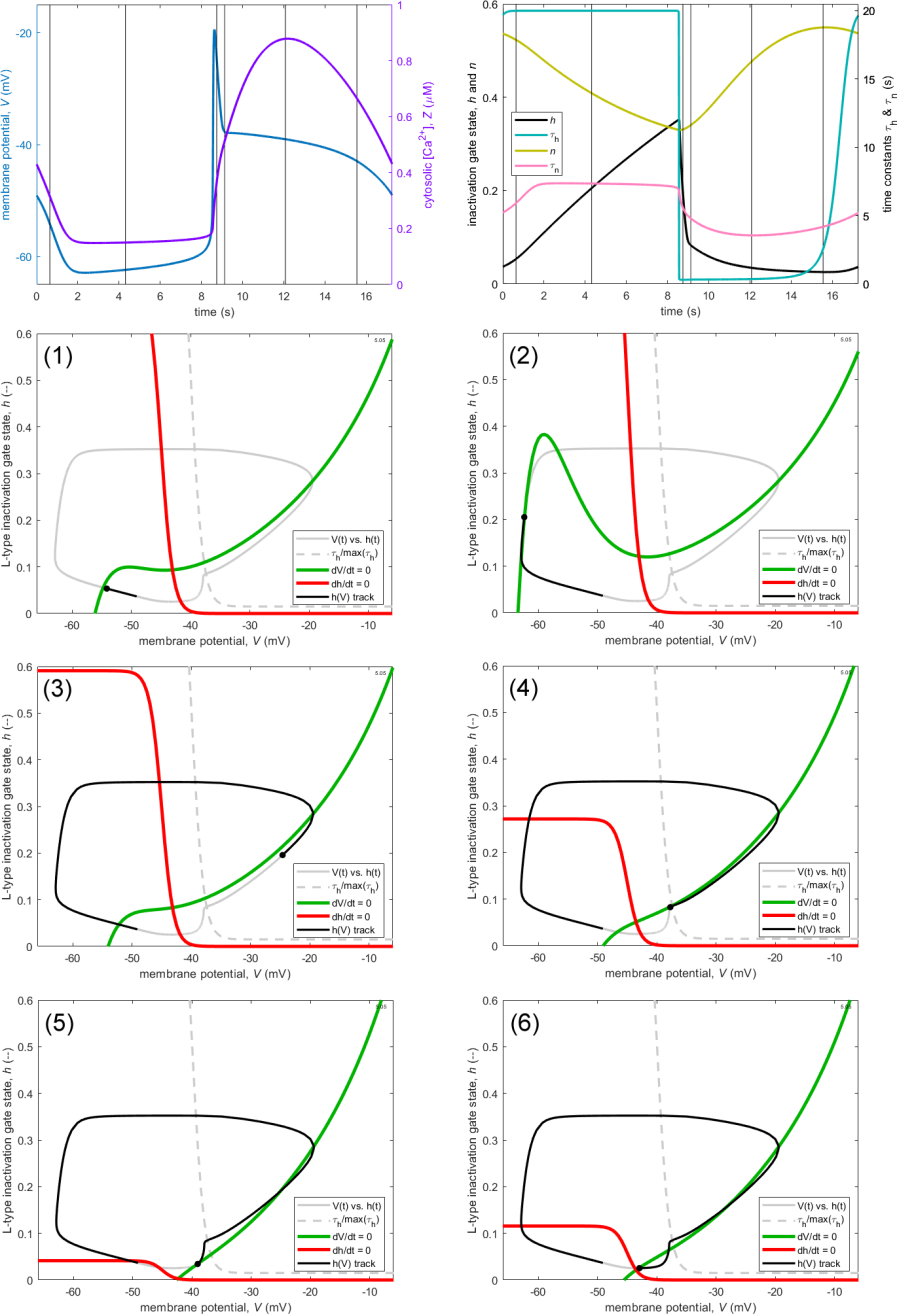
Phase planes as in figure 5 but for the pacemaker oscillation under IP_3_R-KO shown in figure 4, at the six instants shown by vertical lines in the top panels. Since $V_{\text{h}\text{t}}$ here differs from $V_{\text{h}\text{h}}$, the phase planes also show $\tau_{\text{h}}\left(V\right)/\tau_{\text{hx}}$ (dashed).

We now return to the model that incorporates a RyR model and for which the membrane potential dependence for the $\tau_{\text{h}}$ transition is different from that for the h-nullcline, i.e. $V_{\text{h}\text{t}}\neq V_{\text{hh}}$. The form of the relation $\tau_{\text{h}}\left(V\right)$ is indicated by a dashed grey curve added to the (V, h) phase planes in figure 6, which otherwise follows the plan of figure 5 in illustrating six salient times during the oscillation cycle. The starting phase is arbitrary (set to put the AP spike in the centre of the waveform trace), and it will be convenient to discuss the times $t_{1}$ to $t_{6}$ starting at $t_{2}$ and finishing with $t_{1}$.

As before, when the operating point is ascending the left-hand limb of the M-clock orbital trajectory ($t_{2}$), the dynamics of h are slow, despite large $h_{\infty }-h$. Also as before, when the operating point is descending the right-hand limb of the trajectory, the dynamics of h are fast ($t_{3}$), despite somewhat smaller $h_{\infty }-h$, because $\tau_{\text{h}}$ is at or close to its minimum value, now 0.3 s. But the new feature is that Z is sufficiently high (already ≈0.4 µM, and rapidly increasing) that the V-nullcline becomes single-valued in h (cf. $t_{2}$ in figure 5).

By the time the initial spike of the AP has finished, Z is up to ≈0.55 µM, and the lower part of the V-nullcline has moved further to the right ($t_{4}$), such that it is now preventing further decrease of V. The only route open to the trajectory is downwards, towards the h-nullcline. At this point, $h_{\infty }-h$ is relatively small and $\tau_{\text{h}}$ starts to increase (the operating point is in the early part of the $\tau_{\text{h}}$-transition). Therefore dh/dt to decrease, but the major constraint is the V-nullcline, which has met the operating point and therefore slowed dV/dt to almost zero.

Z continues to rise, until by the mid-point of the flatter part of the AP plateau it has reached its maximum value of ≈0.8 µM, causing the V-nullcline to reach its furthest rightward position, and the left-hand part of the h-nullcline to reach its smallest h value ($t_{5}$). With the operating point almost on the V-nullcline, the trajectory is forced to deflect downwards, since dh/dt is not constrained to be quite as small as dV/dt, despite slowly increasing $\tau_{\text{h}}$ and diminishing $h_{\infty }-h$.

Z now descends gradually from its peak, reaching ≈0.66 µM by *t* = 15.5 s, when the AP plateau is almost over ($t_{6}$). The h-nullcline starts to grow again, and the lower part of the V-nullcline retreats very slowly to the left, allowing the orbital trajectory to resume its leftward path. With the h-nullcline growing up past the operating point, h has now reached its minimum.

The transition from the plateau phase to the descending limb of the AP is completed with the V-nullcline resuming its tripartite shape and moving off increasingly rapidly to the left (Z is now down to ≈0.31 µM), with the operating point in pursuit ($t_{1}$). By this time, $h_{\infty }-h$ is large, but $\tau_{\text{h}}$ has completed its own transition to large values, so h can only increase slowly. This mechanism of plateau creation, i.e. blocking by a V-nullcline that becomes monotonically positive in gradient when Z is large ($t_{4}$−$t_{6}$), is also the mechanism responsible for an AP plateau in the model of Imtiaz *et al*. [12], where $h_{\infty }=f\left(Z\right)$ only; see fig. 3 of [10] and the animation that formed supplementary video 1 therein.

### 3.5. Frequency in the absence of a plateau

**Figure 7.**
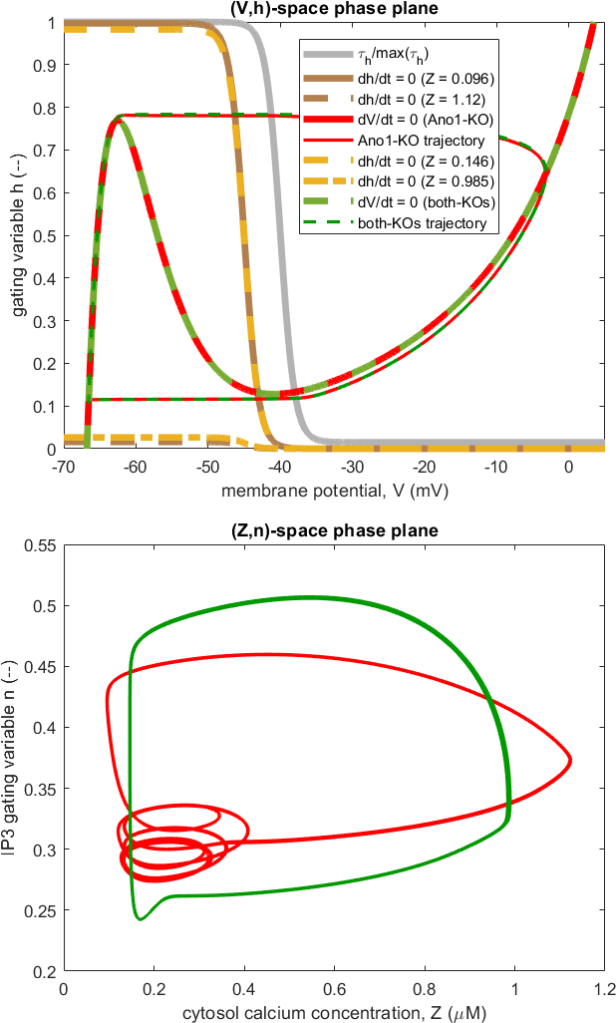
Upper panel: (V, h)-plane trajectories and nullcline extremes under Ano1-KO and combined-KO for the model with RyR channels for which waveforms are shown in figure 4. The trajectory for combined-KO (thin green dashed) overlays that for Ano1-KO (thin red), just as the invariant V-nullcline for combined-KO (thick green dashed) overlays that for Ano1-KO (thick red). The h-nullcline extremes for Ano1-KO (brown) are further apart than those for combined-KO (yellow). Grey: normalized $\tau_{\text{h}}\left(V\right)$. Lower panel: C-clock trajectories for Ano1-KO (red) and combined-KO (green) projected onto the (Z, n)-plane. Many cycles (the last 122.5 s of the simulation) are here superimposed, showing (by localized curve thickening) that the period-6 oscillation varies very slightly with the C-clock phase relative to that of the M-clock.

Why is the period of repetition of combined-KO APs shorter than that of Ano1-KO APs (figure 4)? The difference cannot be explained on the basis of the M-clock alone, which is illustrated in figure 7. The V-nullcline is the same in the two conditions, amounting to that for Z = 0, since Ano1-KO removes the effect of Z on equation (2.4) [10]. There is a large difference in total Z-excursion between the two conditions, which translates into a significant difference in the low-V level of the h-nullcline at the extremes of the Z-excursion (0.096 and 1.12 µM for Ano1-KO, 0.21 and 0.69 µM for combined-KO). But the difference is in the wrong sense to explain why the combined-KO trajectory, which is identical to the Ano1-KO trajectory, is traversed in less time. The lower panel in figure 7 shows how the two conditions compare in terms of (Z, n)-plane trajectories. But here the approximate reduction of the C-clock to two dimensions breaks down, because the two orbits are far apart in terms of the third axis, Y, of the C-clock: Y ranges between 9.7 and 13.1 µM under Ano1-KO and between 33.6 and 36.3 µM under combined-KO, where only leakage prevents store calcium-ion concentration [Ca^2+^]_s_ rising uncontrollably.

The answer relates to the coupling of the M-clock to the much faster C-clock under Ano1-KO, a matter discussed in some detail by Hancock *et al*. [7]—see their fig. 12 and associated description. The C-clock frequency is independent of the M-clock during Ano1-KO, but the size of C-clock Z-peaks is greatly affected by whether an M-clock V-peak, i.e. an AP, occurs simultaneously. Here the coupling necessitates a period-6 oscillation of the C-clock to accommodate the M-clock, whereas the coupled combined-KO oscillation of the C-clock is period-1. The timing of APs is in turn affected by the Z-oscillation, via the Z-dependence in the function $h_{\infty }\left(Z,V\right)$. The small Ano1-KO Z-peak at t = 49 s in figure 4 causes the very small V-peak at the same instant. At *t* = 17 s the much larger combined-KO Z-peak provokes the next combined-KO AP, but at this time, the Ano1-KO Z-peak is too small to allow an AP. The AP is therefore delayed until the time of the next large Z-peak, which is itself amplified by the AP.

As in Hancock *et al*. [7], this delay can be abolished by eliminating the $h_{\infty }\left(Z\right)$-dependence, by setting the parameter $K_{\text{h}}$ to a value substantially higher than occurs at any time in the Ano1-KO Z-trace in figure 4. For the sake of brevity, the result is not illustrated here but is described. The h-nullcline then no longer varies with time but remains constantly at the position indicated by the solid brown curve in figure 7. The M-clock period becomes identical under Ano1-KO and combined-KO. The (V, h)-space trajectory is still as in figure 7, but is traversed faster, because the operating point climbs the left-hand limb of the V-nullcline faster, because $h_{\infty }-h$ is greater. Thus, the now-consensual frequency of both the Ano1-KO and the combined-KO conditions increases to 2.04 Hz, where previously the Ano1-KO frequency was 1.27 Hz and that for the combined-KO was 1.63 Hz. The combined-KO Z-trace continues its period-1 trajectory in the (Z, n)-plane projection of the three-dimensional C-clock phase space, while the Ano1-KO Z-trace takes up a period-4 rhythm.

## 4. Discussion

In this paper, we have presented a novel analysis of circumstances in a model of lymphatic pacemaking that lead to APs with an extended plateau. As in the biological observations shown in figure 1, the model displays the longest-lasting plateaus when its representation of the IP_3_ receptors that release calcium from the intracellular store (the SR) is turned off. The physiological mechanism leading to this association between IP_3_R block and long-lasting APs is not understood at present [19]. Our analysis of this behaviour in terms of the motion of model variables (the experimentally observable membrane potential and a more abstract quantity, the L-type gating variable) on phase planes is intended to offer a fresh perspective on this problem, which may perhaps spark fresh modes of biological investigation.

In the process, we have introduced into our model of LMC pacemaking a ryanodine receptor representation, in parallel with the previously existing IP_3_ receptor representation. In so doing, we have improved the model’s correspondence with observed behaviour under IP_3_R blockade in two important respects: the extent of [Ca^2+^]_c_ excursion when APs occur, and the morphology of the AP itself. Whilst in the previous iteration of our model the IP_3_R-KO AP was similar to that during Ano1-KO, i.e. much taller and shorter than the control AP, now, as noted experimentally, it lasts longer than and is comparable in peak-to-trough excursion with the control AP. And whilst previously the IP_3_R-KO [Ca^2+^]_c_ excursion was almost six times smaller than the control excursion (see fig. 10 of [7]), now the two are similar in magnitude (figure 4), as in fig. 11F of [11], and comparable with calibrated *ex vivo* observations [16].

Finding this solution involved examining the circumstances that must exist in order to create an AP plateau and thereby a long-lasting AP. We identify two distinct dynamical mechanisms that can cause plateau generation. The first, as in figures 3 and 5, is wholly determined within the M-clock, that subset of our coupled-oscillator pacemaker model which creates the AP, whilst, with the introduction of RyRs, the other involves also the C-clock, the model subset behind the oscillation of intracellular [Ca^2+^].

The latter mechanism is illustrated in figures 4 and 6. It rests on the $h_{\infty }=f\left(Z\right)$ part of the $h_{\infty }=f\left(V,Z\right)$ relation driving the V-nullcline so far down and to the right at the time of peak Z that repolarization is prevented until Z decays. The direct involvement of Z is why we term this the C-clock mechanism. It can produce a plateau even if $\tau_{\text{h}}$ is small throughout that part of the cycle as in figure 6 or if the function $\tau_{\text{h}}\left(V\right)$ is arranged with $V_{\text{h}\text{t}}$ substantially less than $V_{\text{h}\text{h}}$ (not shown) to ensure that the phase of prevented repolarization occurs while $\tau_{\text{h}}$ is small.

For the M-clock mechanism of figures 3 and 5, almost all of the plateau occurs after the h-nullcline has dropped down away from the trajectory. The V-nullcline also retains two turning points, remaining cubic throughout. This implies that the plateau is primarily being driven by the varying parameter $\tau_{\text{h}}\left(V\right)$ and not by $h_{\infty }-h$ and consequently by Z. It is not a pure M-clock mechanism, but the plateau is predominantly driven by the M-clock in this case.

In addition, we find it advantageous for the case shown in figures 4 and 6 to set the $\tau_{\text{h}}\left(V\right)$ transition to occur at a higher value of V than the $h_{\infty }\left(V\right)$ transition itself, i.e. to set *V*_ht_ > *V*_hh_. This means that the dynamics of h, already slow because $h_{\infty }-h$ is small, are slowing further during the plateau, introducing the slowly executed ‘dog-leg’ into the (V, h) trajectory. In another case, not illustrated here, the separation of $V_{\text{h}\text{h}}$ and $V_{\text{h}\text{t}}$ also allowed us to create a control AP that included a notch (a local minimum) immediately after the initial spike, a feature that is sometimes seen *in vivo*, albeit not in figure 1. However, the notch was achieved using a set of parameter values that caused the IP_3_R-KO AP to revert to the plateau-less shape of the Ano1-KO AP, emphasizing the likely impossibility of simultaneously satisfying all goals with a simplified model.

**Figure 8.**
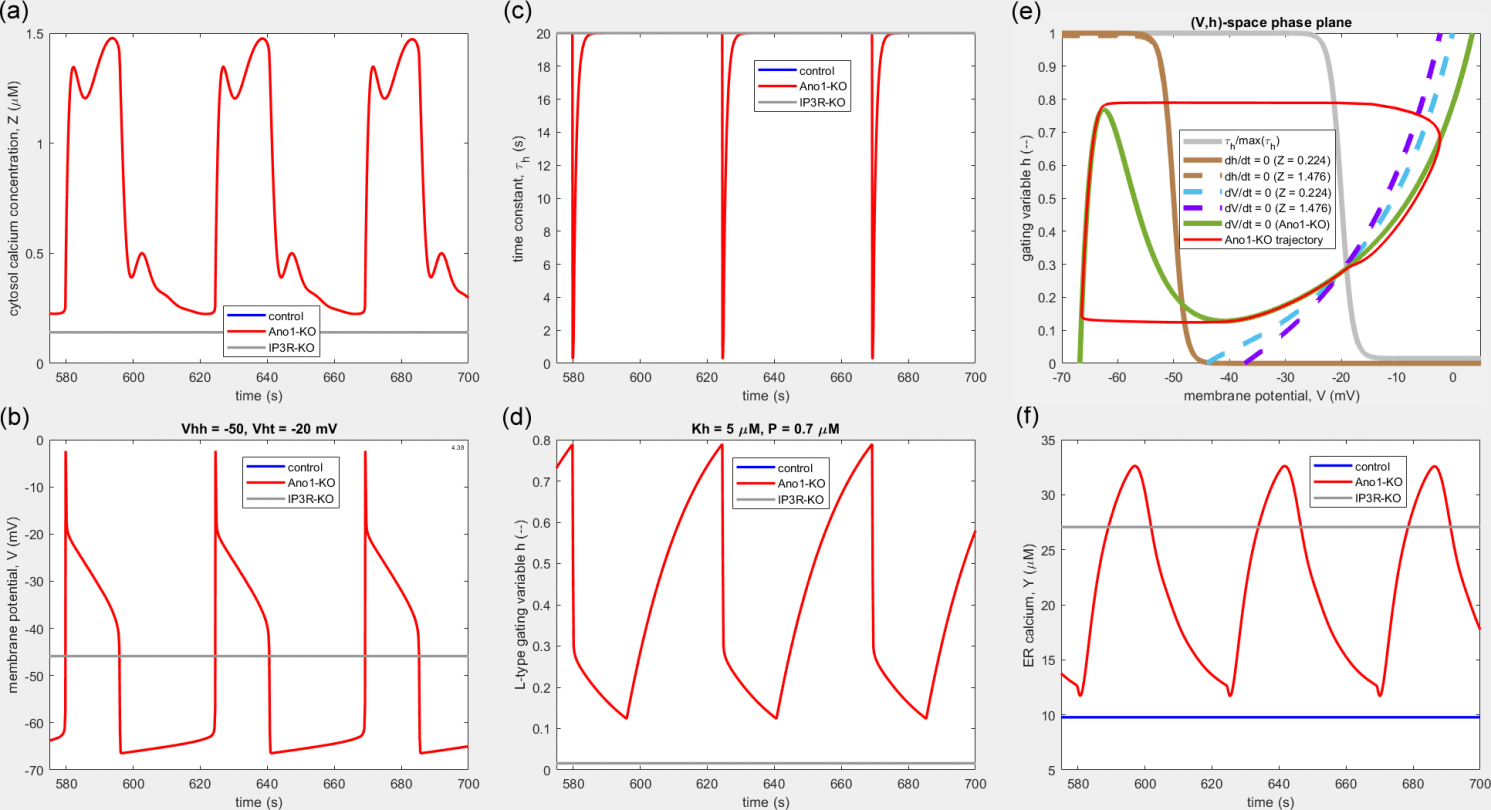
Waveforms and the (V, h)-phase space for an example of the model as configured for figures 3 and 5, i.e. without the RyR but with $V_{\text{h}\text{t}}\gg V_{\text{hh}}$ and other parameter value adjustments. Only the Ano1-KO condition (red) leads to pacemaking; control (blue) and IP_3_R-KO (grey) conditions settle to an unchanging state. The waveforms are (*a*) $Z\left(t\right)$, (*b*) $V\left(t\right)$, (*c*) $\tau_{\text{h}}\left(t\right)$, (*d*) $h\left(t\right)$ and (*f*) $Y\left(t\right)$; except in (*f*), the grey line overplots the blue one. The IP_3_ gating variable n (not shown) executes a waveform similar in shape and phase to $Y\left(t\right)$, between extremes of 0.322 and 0.729. In the phase plane (*e*), the h-nullcline (brown) is shown at its extremes corresponding to the minimum (solid line) and maximum (dashed line) values of $Z\left(t\right)$. Curves are shown for a V-nullcline at these same values (dashed blue and purple), but under Ano1-KO, the V-nullcline is fixed (green) independently of $Z\left(t\right)$. Grey: normalized $\tau_{\text{h}}\left(V\right)$. Parameter values differ from those used in figure 4 and listed in table 1 as follows: $J_{\text{xce}}$ = 0.56$v_{c}/b_{c}$ µmol s^−1^, $K_{\text{C}}$ = 0.25 µM, $G_{\text{xC}}$ = 0.15 nS, q = 1, $K_{\text{h}}$ = 5 µM, $V_{\text{hh}}$ = −50 mV, $V_{\text{h}\text{t}}$ = −20 mV.

The benefit of setting *V*_ht_ > *V*_hh_ can be further illustrated and understood by means of an example in which the difference is exaggerated, i.e. $V_{\text{h}\text{t}}\gg V_{hh}$. With parameters as set for figure 8, only the case of Ano1-KO oscillates (the model reaches a stable equilibrium for control and IP_3_R-KO conditions), but now the Ano1-KO AP includes a prominent (sloping) plateau. In complete contrast to all previous Ano1-KO APs shown, the AP duration is now much longer: more than one-third of the total cycle time. With $V_{\text{h}\text{t}}$ set to −20 mV, the rapid decay of the AP spike is suddenly arrested at this level, when, with $\tau_{\text{h}}$ now arriving back at its maximum value of 20 s, dh/dt is abruptly reduced. This causes h to have to crawl slowly down to the value corresponding to the minimum of the V-nullcline, when the rapid dynamics of V take over for the horizontal translation across to the left arm of the V-nullcline, completing the repolarization.

It may be argued that a limitation of this study is that we have assumed parameter values as needed to produce the phenomena under consideration. The parameter values assumed here may not be feasible physiologically. In particular, we have assumed that the time course of L-type channel inactivation via the gating variable h is a very strong function of V, varying over a 67-fold range. However, rather similar behaviour is seen in established models. In the L-type channel model of Jafri *et al*. [25] for cardiac ventricular myocytes, the extremes of $\tau_{\text{h}}\left(V\right)$ are 620 and 20 ms, a 31-fold change, with the transition mid-point occurring at −30 mV. Furthermore, in our deliberately simplified model, in which the AP is largely caused by Ca^2+^ fluxes, this function has a dual purpose, also allowing a rapid AP upstroke spike, something that biologically may also involve voltage-gated Na^+^ channels [26]. We have also assumed, in allowing separate constants $V_{\text{h}\text{h}}$ and $V_{\text{h}\text{t}}$, that the functions $h_{\infty }\left(V\right)$ and $\tau_{\text{h}}\left(V\right)$ can transition from high to low values at differing values of V. To our knowledge, this possibility has not been examined by biologists thus far, but the gate function providing voltage-dependent inactivation in the model of Jafri *et al*. [25] is half closed at −55 mV (i.e. their *V*_ht_ > *V*_hh_). Finally, the purpose of this paper is qualitative: to demonstrate that two dynamical mechanisms of plateau formation exist. We have therefore chosen parameter values to optimize these demonstrations, without undue concern for their physiological veracity. However, they are based on existing models and experiments, as indicated in table 1.

A further limitation is that the model is greatly simplified. The concept of such a model involves accepting many approximations that would be ruled out in a fully detailed model. Thus, for instance, Ca^2+^ is the only ion that is fully budgeted in our model, whereas other ions (Na^+^, K^+^ and Cl^−^) are involved biologically. Even the calcium equations are simplified: for instance, equation (2.7) makes Ca^2+^ influx from outside exclusively a function of intracellular [IP_3_]. This non-dependence of $J_{\text{ci}}$ on V is inherited from the model of Imtiaz *et al*. [12], which provided the original inspiration for all varieties of our model developed so far. We continue to explore the limits of what a simplified model can show, modifying it only as much as the biological data compel, believing that such models can provide enlightenment where fully detailed models may obscure or confuse.

## 5. Conclusion

By incorporation of a ryanodine receptor model, we have greatly improved the extent to which our previously developed model of pacemaking agrees with recent experimental findings on the rhythmic generation of action potentials and calcium flashes in lymphatic muscle cells, under four different experimental conditions. We have also presented a novel detailed analysis of two distinct dynamical mechanisms by which the model predicts a systolic plateau in the action potential under two of these conditions, mimicking what is observed. The model consists of two coupled oscillators or clocks, one centred on cellular electrical events and the other on calcium ion exchange between the cell and an internal store, and each mechanism relates to one of these clocks. In summary, this paper provides a simplified model with representative parameter values that together provide good qualitative agreement with experimental observation, along with phase plane interpretations for the mechanisms involved, thus providing an excellent starting point for future data-fitting studies. The modelling has the potential to help devise means of intervention in lymphatic diseases involving deficits in the active pumping of lymph.

## Data Availability

Matlab code for solving the equations is accessible at the Sydney eScholarship Repository (https://hdl.handle.net/2123/32566). Supplementary material is available online [27].

## Footnotes

^1^
These data confirm an earlier measurement [17] that showed [Ca^2+^]_c_ moving between trough and peak values of 0.12 and 0.24 µM.

^2^
As before [7] and as is standard practice [18], *J*_ce_ is excluded from equation (2.4) because of the counter-acting pumping of H^+^ ions.

^3^
Biologically, [IP_3_] varies on a slower time scale than that of the rhythmic pacemaking we consider here.

^4^
Initial spike height is very sensitively affected by the nature of the ‘stick’, i.e. the impalement of the LMC by the electrode via which the membrane potential is recorded. In general, we find that better-quality impalements lead to larger initial spikes. In some cases, the initial spike under control conditions can momentarily almost double the height of the upstroke of the AP.

^5^
The time-varying time constant *τ*_n_ is not explicit in equations (2.1)−(2.23); it arises from rearranging equation (2.3) into a form equivalent to equation (2.5) and solving for *τ*_n_. See Hille [24].

^6^
This excludes the negative-slope portion of the *V*-nullcline, which equivalently repels instead.
